# Progress in Obstetrics and Gynæcology

**Published:** 1875-01

**Authors:** A. Reeves Jackson

**Affiliations:** Lecturer on Gynæcology, Rush Medical College, Chicago


### Progress in Medical Sciences.

ARTICLE I.

### PROGRESS IN OBSTETRICS AND GYNÆCOLOGY.

BY A. REEVES JACKSON, M.D.,
LECTURER ON GYNÆCOLOGY, RUSH MEDICAL COLLEGE, CHICAGO.

1. Puerperal Pyæmia. By Dr. J. MATHEWS DUNCAN. (*Obstetrical Journal of Great Britain and Ireland*, September, 1874.)

2. On the Natural and Artificial Elimination of Sessile (Intra-Parietal Uterine Fibroids. By Dr. MANNEL. (*Amer. Journal of Obstetrics*, November, 1874. *Viertel jahrschr. fur die prakt. Heilk.*, 1874.)

3. Case of Uterine Fibroid removed according to a New Principle of Operation. By Dr. T. ADDIS EMMET. (*Amer. Journal of Obstetrics*, August, 1874.)

4. Intra-Uterine Fibroids. By Dr. J. MARION SIMS. (*New York Medical Journal*, April, 1874.)

1. In the course of an address delivered at the annual meeting of the British Medical Association, in August last, Dr. Duncan stated that for obstetricians, and for the world, the subject of puerperal fever was the one of first importance in midwifery, since there was unanimity in giving it the horrid pre-eminence over all other combined causes of death in the child-bed or puerperal state. He objects, however, to the term puerperal fever, as one embodying error. There is nothing, he contends, essentially puerperal known in it; nor is there anything about it of the nature of a fever, as that word is generally understood. He prefers the term pyæmia, as being most in accord with present views of pathology, although advancing science may replace it by a better one. Pyæmia is not to be understood, however, as meaning purulent blood—its former signification—but as a comprehensive term including septicæmia, icorrhæmia, etc. There is no proof that puerperal pyæmia is ever epidemic as fevers are. Neither is it caused by a miasma, for even in a pestilential hospital, the disease prevails in such a manner as is scarcely reconcilable with the miasma hypothesis. Likewise, Dr. Duncan does not believe that the disease is propagated by contagion or infection, and, in this connection, he ridicules the prevalent idea that a practitioner who is so unfortunate as to have a case of the so-called puerperal fever, must therefore relinquish his obstetrical practice. Nevertheless, he warns the obstetrician against assuming any of the duties of the nurse, and thinks that by observing this caution, the medical attendant may make himself medically clean in hands, person and dress.

Our author denies that puerperal pyæmia is, in any sense, a kind of cholera, or typhus, or scarlatina, or erysipelas, as various authors have endeavored to show. On the contrary, he believes that the important researches of Lister and others—especially Lister—demonstrate that pyæmia is a septic disease, and that in its puerperal form it may be almost, if not altogether, prevented by a midwifery practice based on antiseptic principles, combined with stoppage of injurious communication between the sick and the healthy, disinfection, and such architectural, and other arrangements in lying-in hospitals, as may secure the fullest and best hygienic conditions.

There can be no doubt as to the great importance of the subject of Dr. Duncan's paper, but there is much doubt whether his opinions will be adopted as correct by the great body of the medical profession.

In the most favorable cases of parturition there is almost inevitably more or less wounding of the generative passages, and these contusions and lacerations present the most accommodating localities for the reception of morbific material. A woman who is the subject of puerperal pyæmia becomes a prolific source of morbific material in its most subtle and essential form. That puerperal pyæmia is frequently communicated by conveyance of this septic essence—whatever it may be—from the person of one patient to that of another, with the result of reproducing the disease, has been too frequently demonstrated to admit of doubt; and hence, the obstetrician cannot be too careful to protect his patients from this source of danger.

2. Dr. Mannel thinks that the absorption of uterine fibroids by the administration of medicines does not occur, and that when such results have been reported, the diagnosis was erroneous, the cases being hyperplasia of the uterus, or plastic exudation in its vicinity. He regards Hildebrandt's treatment of these growths by the hypodermic injection of ergotine as needing further trial in order to determine its value. At present he evidently attaches but little value to it. The induction of sloughing of the tumor by any means, mechanical or chemical, he considers dangerous, and as too likely to result fatally from pyæmia, septicæmia, peritonitis, venous thrombosis, or exhaustion. The paper is written chiefly for the purpose of showing the results of enucleation—the only surgical proceeding of which he approves. He has collected twenty-two cases, more or less in detail, in which enucleation has been employed for the removal of uterine fibroids since the year 1858. The table is supplementary to that of Dr. West, who reported twenty-seven cases down to the year named. Of Mannel's cases only three terminated fatally,—a very favorable percentage; while of West's twenty-seven cases, fourteen were fatal. Thus, of forty-nine cases there were thirty-two recoveries and seventeen deaths.

Unfortunately for the usefulness of statistics such as the foregoing, it is well known that many unsuccessful cases are never reported, while those which result favorably are very likely to be.

3. In Dr. Emmet's case, the patient had been suffering for a month with excessive metrorrhagia, which had been kept in check only by the continued use of powerful styptics. The cavity of the uterus was found to be occupied by a fibrous tumor of the size of the fist. Suppositories of gelatine, each containing 16 grs. Squibb's aqueous extract of ergot (equivalent to 96 grs. powdered ergot) were introduced into the cavity of the uterus, where they produced a markedly beneficial result. The tumor, which was attached by a broad base to the fundus, was brought near to the internal os. Dr. Emmet retroverted the uterus, which had been previously anteverted, and then seizing the tumor with a double tenaculum, drew it downwards toward the vulva, to which point he succeeded in bringing it after half an hour's steady traction. Portions of the tumor as it became attainable were removed by scissors. As the tumor was drawn down, the uterus contracted behind it, thus by its own efforts enucleating the base of the growth, and at the same time preventing hemorrhage. This made it necessary only to divide the capsule of the tumor with the scissors in order to remove the whole growth. The base of the tumor measured about two inches in diameter.

After its removal, Dr. Emmet followed his usual custom of washing out the uterus with warm water, and then painting the whole of its cavity with Churchill's tincture of iodine, as a precaution against septicæmia.

In speaking of the *rationale* of the operation, Dr. Emmet stated that he thought the steady traction arrests and prevents hemorrhage by exciting the uterus to contract behind the tumor and thus to compress the bleeding vessels; and that although the traction brings the tumor nearer and more convenient for removal, yet that it is the contraction of the uterine wall which is the efficient agent in lifting the tumor from its bed and enucleating it.

4. In this paper Dr. Sims details eight cases of large intra-mural fibroids, in six of which the tumors were successfully removed. Of the other two, one died from peritonitis, after the use of a sponge tent introduced as a preparatory measure, and the other barely escaped with her life from the constitutional disturbance produced by the same cause.

Dr. Sims enunciates two guiding principles to be observed in the artificial removal of intra-uterine fibroids: 1. The cervical canal must be freely opened. 2. The tumor must be freed from the restraint of its investing capsule.

In detail, his plan of operating is as follows: The patient must be placed under the best hygienic conditions, and then, the cervix having been widely opened the previous night by as many sponge tents as can be introduced at one time, the patient is placed in the semi-prone position and the vagina opened with a Sims speculum. The presenting portion of the tumor is seized with a strong vulsellum, and pulled forward, and its capsule opened with scissors, cutting squarely into it, and taking care not to separate the capsule from the cervix. The finger is then passed into the opening thus made, between the the tumor and its capsule. This latter should be left attached to the walls of the uterus, but should be divided all around in close proximity to the borders of the cervix. The tumor being firmly held by the vulsellum, a rectangular hook, called an enucleator, is pushed rapidly up between the tumor and its capsule as far as the fundus on every side, and swept around the tumor until all the cellular and fibrous tissues connecting it with its capsule are severed. This having been accomplished, a strong double hook is passed up behind the tumor as far as possible into the cavity of the uterus, and, grasping the tumor, the latter is pulled down and slightly rotated on its vertical axis, the enucleator still pursuing its work of detachment. As the tumor comes down, the hook is passed farther up and fastened again, and thus the growth is finally brought outside the vulva. After the removal of the tumor, loose shreds are cut away with scissors, and the uterine cavity firmly plugged with iron-cotton for twenty-four hours. Should fetid discharge and septic symptoms appear, the cavity of the uterus should be freely washed out with warm, carbolized water.

From the foregoing articles on uterine fibroids, one may gather the present drift of treatment in this disease. It is evidently toward the most radical means for removal. And yet there has never been a time when such means seemed less necessary. Hildebrandt, and many others, have reported cases (now quite numerous) in which uterine fibromata of large size have either entirely disappeared or been so reduced in size as to render them comparatively innocuous, by the persistent use of ergotine alone, given hypodermically or otherwise. And it is presumptuous, if not absurd, to say, in answer to these reports, that the cases were uterine hyperplasia or perimetric inflammatory exudation, and not uterine fibroids at all, as has been done by Dr. Mannel.

While it is unquestionably true that many uterine fibroids may be safely removed by surgical procedures—and we regard the plan pursued by Drs. Sims and Emmet as the best for sessile growths—it is equally true that in many others operative measures are exceedingly dangerous. Hence, although the great degree of success claimed by Dr. Hildebrandt has not perhaps been equaled by others who have made trial of the ergotine treatment, yet in almost every suitable case in which the use of the drug has been persisted in for a sufficiently long time, there has been marked improvement in the general health of the patient, hemorrhage has been lessened or entirely checked, and pain has abated or disappeared.

In the present state of our knowledge of this subject, we regard the surgical removal of intra-uterine sessile fibroids as imprudent and improper, unless a dangerous amount of hemorrhage, pain, or exhaustion remain after a full trial of the ergotine treatment; for it is only the presence of these symptoms that justifies or necessitates any interference with such growths.

